# Impact of genotype 1 and 2 of porcine reproductive and respiratory syndrome viruses on interferon-α responses by plasmacytoid dendritic cells

**DOI:** 10.1186/1297-9716-44-33

**Published:** 2013-05-15

**Authors:** Arnaud Baumann, Enric Mateu, Michael P Murtaugh, Artur Summerfield

**Affiliations:** 1 Institute of Virology and Immunoprophylaxis (IVI), Sensemattstrasse 293, Mittelhäusern, 3147, Switzerland; 2 Graduate School for Cellular and Biomedical Sciences, University of Bern, Bern, Switzerland; 3 Centre de Recerca en Sanitat Animal (CReSA), UAB-IRTA, Campus de la Universitat Autònoma de Barcelona, Barcelona, Bellaterra, 08193, Spain; 4 Department of Veterinary and Biomedical Sciences, University of Minnesota, St. Paul, MN, 55108, USA

## Abstract

Porcine reproductive and respiratory syndrome (PRRS) virus (PRRSV) infections are characterized by prolonged viremia and viral shedding consistent with incomplete immunity. Type I interferons (IFN) are essential for mounting efficient antiviral innate and adaptive immune responses, but in a recent study, North American PRRSV genotype 2 isolates did not induce, or even strongly inhibited, IFN-α in plasmacytoid dendritic cells (pDC), representing “professional IFN-α-producing cells”. Since inhibition of IFN-α expression might initiate PRRSV pathogenesis, we further characterized PRRSV effects and host modifying factors on IFN-α responses of pDC. Surprisingly, a variety of type 1 and type 2 PRRSV directly stimulated IFN-α secretion by pDC. The effect did not require live virus and was mediated through the TLR7 pathway. Furthermore, both IFN-γ and IL-4 significantly enhanced the pDC production of IFN-α in response to PRRSV exposure. PRRSV inhibition of IFN-α responses from enriched pDC stimulated by CpG oligodeoxynucleotides was weak or absent. VR-2332, the prototype genotype 2 PRRSV, only suppressed the responses by 34%, and the highest level of suppression (51%) was induced by a Chinese highly pathogenic PRRSV isolate. Taken together, these findings demonstrate that pDC respond to PRRSV and suggest that suppressive activities on pDC, if any, are moderate and strain-dependent. Thus, pDC may be a source of systemic IFN-α responses reported in PRRSV-infected animals, further contributing to the puzzling immunopathogenesis of PRRS.

## Introduction

Type I interferons (IFN), mainly IFN-α/β, are essential to the innate immune system for direct antiviral activity as well as efficient induction of adaptive immune responses [1,2]. This critical role is underlined by the fact that seemingly all viral pathogens have evolved strategies to counteract this innate defense system [3].

Porcine reproductive and respiratory syndrome (PRRS) virus (PRRSV), an enveloped positive-sense, single-stranded RNA virus, has been associated with a low innate and delayed adaptive immune response [4]. The virus is characterized by an enormous genetic variability with the existence of two genotypes of PRRSV referred as genotype 1 (European) and 2 (North American), and the emergence of highly virulent isolates in Asia within genotype 2 [5]. Macrophages in lung and lympoid tissues are the primary site of PRRSV replication [6,7], although other cell types such as monocyte-derived dendritic cells (MoDC) and monocyte-derived macrophages are susceptible to infection [8,9]. Due to the persistence of PRRSV in infected pigs, it was proposed that the virus modulates host innate and acquired immune responses [10,11]. While PRRSV is highly sensitive to IFN-α both in vitro [12-14] and in vivo [15], the virus promotes weakly or not at all in vitro synthesis of type I IFN in porcine alveolar macrophages (PAM) and MoDC [16-18]. However, systemic IFN-α was observed after infections with various PRRSV isolates [15,16,19,20], indicating that certain cell types are able to sense infection.

Plasmacytoid dendritic cells (pDC) are a major source of IFN-α and other inflammatory cytokines after exposure to TLR7 and TLR9 ligands, including many viruses and bacterial DNA [21]. Although pDC are a rare cell type, they can produce around 100 times more IFN-α than any other cellular type. They are often able to sense viruses in the absence of viral replication. Consequently, they represent an important candidate cell type for investigating early immune events that could influence early control of virus replication or induction of adaptive antiviral immune response [22]. In the pig, these cells have been identified as CD4^+^CD123^+^CD135^+^CD172a^+^CD14^-^, which can be differentiated from monocytes, macrophages and MoDC which lack CD4, CD123 and CD135 but express CD14 and in the case of macrophages and monocyte subset also CD163 [23,24]. Interestingly, stimulation of pDC with genotype 2 PRRSV was reported not to result in detectable IFN-α release [25]. Moreover, the IFN-α production induced by CpG oligodeoxynucleotides (ODN) or transmissible gastroenteritis virus (TGEV) was potently inhibited by North American PRRSV [26]. Considering the observation of in vivo IFN-α, important differences in the virulence of genotype 1 and genotype 2, and the possible regulation of pDC responses by cytokines induced during PRRS infection, we examined how PRRSV of different genotypes and virulence interact with pDC and how cytokines influence pDC responses.

## Material and methods

### Viruses

For the European genotype 1 PRRSV we used Lelystad virus (LV; kindly obtained from Dr Gert Wensvoort, Central Veterinary Institute, Lelystad, The Netherlands) [27] and its counterpart adapted to grow in MARC-145 (LVP23; kindly obtained from Dr Barbara Thür, IVI, Switzerland), 2982, 3267 [28] and Olot/91 (passaged several times, kindly obtained from the PoRRSCon Consortium through Dr Luis Enjuanes, Universidad Autónoma, Madrid, Spain). For the genotype 2 PRRSV we employed the prototype VR-2332 [29] (ATCC, LGC Standards, Molsheim, France), SS144, MN184, JA-1262 and SY0608 (kindly obtained from Dr Martin Beer, Friedrich-Loeffler-Institut, Riems, Germany) representing a highly pathogenic field isolate in China from 2006 [30]. The PRRSV isolate SS144 is from a severe reproductive and respiratory outbreak with high levels of mortality in a previously PRRSV-negative herd in 2010, Missouri, USA. The MN184 isolate is from a farm experiencing severe reproductive disease and sow mortality in 2001, Minnesota, USA. The JA-1262 isolate was obtained in 2009 from a midwestern USA sow herd experiencing abortions and PRRSV-infected weaning piglets. Viral stocks of LV, 2982 and 3267 Spanish field isolates, MN184 and SS144 were propagated in PAM. Strains of Olot/91, LVP23 representing LV adapted to grow in MARC-145 cells after 23 passages, SY0608, JA-1262 and prototype VR-2332 were propagated in the MARC-145 cell line. Cells were lysed by freezing when 50% cytopathic effect (CPE) was reached, clarified by 2500 *g* centrifugation at 4°C for 15 min, and frozen at −70°C until use. Lysates from PAM or MARC-145 cells were used as mock-infected controls. All strains were titrated in their corresponding propagating cell type by CPE evaluation or by using the immunoperoxidase monolayer assay (IPMA) with PRRSV anti-nucleocapsid monoclonal antibodies (mAb) SDOW17-A or SR30-A (Rural Technology Inc., South Dakota, USA). Titers were calculated and expressed as 50% tissue culture infective dose per mL (TCID_50_/mL).

### Cells and pDC enrichment

**Figure 1:**
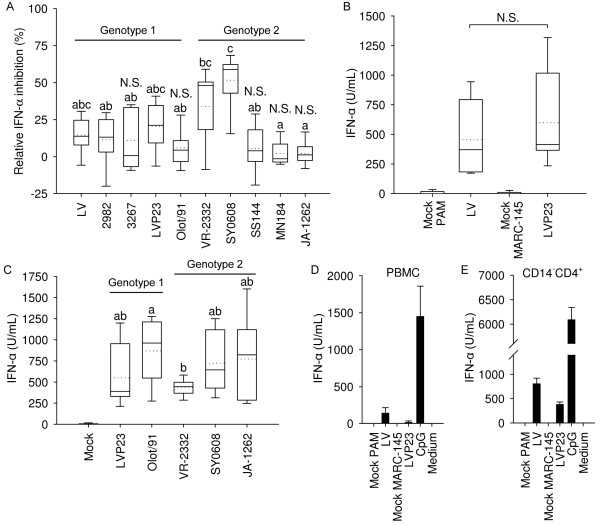
**Effects of PRRSV genotype 1 and 2 isolates on IFN**-**α responses of enriched pDC.** (**A**) PRRSV impact on CpG-induced IFN-α production by pDC. Enriched pDC were stimulated with CpG in presence of the indicated PRRSV isolates added at an MOI of 0.1 TCID_50_/cell. (**B**) Induction of IFN-α by prototype 1 LV and LVP23 (MOI of 1 TCID_50_/cell) in CD172a-enriched pDC (**C**) Comparative analysis of pDC IFN-α responses induced by various PRRSV strains (MOI of 1 TCID_50_/cell). (**D**-**E**) PBMC (D) and CD14^-^CD4^+^ monocyte-depleted enriched pDC (**E**) stimulated with the prototype of genotype 1 LV, LVP23 and CpG-ODN as control. IFN-α was determined by ELISA in supernatants harvested after 20 h. Boxplots in A, B and C indicate the median (middle line), 25^th^ and 75^th^ percentiles (boxes), maximum and minimum (whiskers) and the mean values (dotted line) calculated from at least three independent experiments with cells from different animals each performed in culture triplicates. Bars in (**D**) and (**E**) indicate culture triplicates ± 1 standard deviation. One of two representative experiments is shown. For (**A**) and (**C**), significance between isolates are indicated by different letters based on an ANOVA on Ranks and Dunn's Method pairwise multiple comparison (*P* < 0.05). In (**A**) not statistically significant suppression compared to mock-treated cells was determined by Mann–Whitney Rank Sum test (*P* < 0.02) and noted N.S. = not significant.

MARC-145 cells (ATCC, LGC Standards, Molsheim, France) were grown in Dulbecco’s modified Eagle’s medium (DMEM; Gibco, Invitrogen, Switzerland) supplemented with 10% fetal bovine serum (FBS; Biowest, France). PAM were obtained from bronchoalveolar lung lavages [31]. Specific-free pathogen (SPF) pigs from 6 week- to 12 month-old were euthanized and lungs were aseptically removed. Briefly, lungs were filled up with approximately one to two liters of PBS containing a 2× concentrated penicillin/streptomycin (Pen/Strep) solution (Gibco, Invitrogen, Switzerland). The lavage was collected and cells were recovered by centrifugation (350 *g*, 10 min, 4°C), followed by three wash steps with 2 × Pen/Strep PBS and centrifugation at 350 *g* for 10 min. PAM were maintained in RPMI 1640 medium (Gibco) supplemented with Pen/Strep and 10% FBS or frozen in liquid nitrogen until use. MARC-145 cells and PAM were cultured at 37°C in a 5% CO_2_ atmosphere. MoDC were prepared using interleukin-4 (IL-4)/granulocyte–macrophage colony-stimulating factor (GM-CSF) as previously described [32]. For all experiment except in Figure 1D and 1E, CD172a enrichment of pDC was performed as described earlier [33]. Briefly, peripheral blood mononuclear cells (PBMC) from 6 week- to 12 month-old pigs were isolated by Ficoll-Paque differential centrifugation [34] followed by CD172a (mAb 74-22-15a) enrichment using MACS sorting LD columns (Miltenyi Biotec GmbH, Germany) leading to > 80% of CD172a positive cells and 2-8% CD172^low^CD4^high^ pDC. The cells were cultured in DMEM with 10% FBS and 20 μM of β-mercaptoethanol (Invitrogen, Switzerland). For the experiment shown in Figure 1D and 1E, PBMC were depleted of monocytes by anti-CD14 (mAb CAM36A, VMRD Inc., Washington, USA) followed by CD4 (mAb PT90A, VMRD Inc.) selection with MACS sorting LS column (Miltenyi Biotec GmbH, Germany). This sorting resulted in a pDC purity of 10%.

### Stimulation of pDC and IFN-α ELISA

Enriched pDC were incubated at 400’000 per microwell with CpG-ODN D32 [33] (10 μg/mL; Biosource Int., Camarillo, USA) and PRRSV strains at a multiplicity of infection (MOI) of 0.1 to 2.5. Inactivation of PRRSV was performed in a UV chamber (Biorad; GS Gene Linker) at 100 mJ on ice. Virus inactivation was verified in MARC-145 cultures. Cytokine and other treatments included IFN-β (100 U/mL [35]), IFN-γ (10 ng/mL, R&D Systems, UK), Flt3-L (100 U/mL [24]), GM-CSF (100 U/mL [36]), IL-4 (100 U/mL [32]), TLR7 inhibitor IRS661 (5′-TGCTTGCAAGCTTGCAAGCA-3′, Biosource Int. Camarillo, USA), and TLR7 agonist R837 (10 μg/mL; Biosource Int., Camarillo, USA, [37]). Secreted IFN-α after 20 h of incubation was measured by ELISA as described [38]. The relative proportion of stimulation or inhibition was calculated as the absolute percentage of 100 - ((IFN-α produced by PRRSV treated cells + CpG-ODN)/(IFN-α produced by mock-treated cells + CpG-ODN) · 100).

### Detection of PRRSV antigen and intracellular IFN-α by flow cytometry

To detect PRRSV replication, two million CD172a^+^ cells were seeded in 24-well plates and infected with LV, 2982 and 3267 PRRSV strains at an MOI of 2.5 TCID_50_/cell. Cells were incubated for 24, 48 and 72 h and supernatants tested for IFN-α by ELISA. At each time point the cells were analyzed by three-color flow cytometry for expression of CD172a, CD4 and viral nucleocapsid protein. After staining with the cell surface marker followed by goat isotype specific anti-mouse fluorescein isothiocyanate (FITC) or R-phycoerythrin (RPE) conjugates (SouthernBiotech, Birmingham, AL, USA), the cells were fixed with 4% paraformaldehyde, washed and permeabilized with 0.3% (wt/vol) saponin in PBS. The anti-nucleocapsid mAb SDOW-17A was added during the permeabilization step for 15 min, followed by a wash step with 0.1% (wt/vol) saponin and addition of biotinylated goat anti-mouse IgG1 conjugate (SouthernBiotech), diluted in 0.3% (wt/vol) saponin for 20 min at 4°C. After washing, Streptavidin SpectralRed® (SouthernBiotech) was added as fluorochrome for the FL3 channel. Electronic gating based on the forward/side scatter plots were applied to identify living cells and pDC were defined as CD172^low^CD4^high^ and monocytes as CD172^high^CD4^neg^ population [23]. For intracellular IFN-α staining, one million CD172a^+^ cells were seeded in a 48-well plate and infected with LVP23 strain at an MOI of 1 TCID_50_/cell. After 12 h of culture, Brefeledin A (eBioscience, Austria) was added to the cells to block IFN-α secretion for 4 h. As positive control cells were stimulated with CpG-ODN for 2 h and incubated with Brefeldin A for further 4 h. Cells were then stained for surface CD4 and CD172a markers as mentioned above. Cell fixation and permeabilization for intracellular staining of IFN-α was performed with the Fix & Perm kit (Caltag, UK). Anti-IFN-α mAb F17 (0.3 μg/mL; R&D Systems), biotinylated goat anti-mouse IgG1 conjugate (SouthernBiotech) and Streptavidin SpectralRed® (SouthernBiotech) were added to the cells to detect intracellular IFN-α. The data were acquired using a FACScalibur and analysed using CellQuest Pro Software (BD Biosciences, Mountain View, CA, USA).

### Statistical analysis

Data were analyzed by SigmaPlot 11.0 software. Significant differences between groups were assessed by the Kruskal-Wallis One Way Analysis of Variance (ANOVA on Ranks) and Dunn's Method pairwise multiple comparison (*P* < 0.05 was considered significant). For significance of cytokine-enhancement experiment, the Mann–Whitney Rank Sum test was employed (*P* < 0.02).

## Results

### No or weak suppression of IFN-α in enriched pDC by various strains of PRRSV

Considering the reported suppressive activity of PRRSV genotype 2 isolates on pDC activation [26], we compared the ability of virulent type 1 and type 2 PRRSV to suppress potent IFN-α induction by CpG-ODN. To this end, we simultaneously exposed enriched pDC to various PRRSV strains and CpG-ODN for 20 h and measured IFN-α in the supernatants. The highly pathogenic Chinese type 2 isolate SY0608 showed the highest inhibitory effect at 52% and was the only isolate with inhibitory activity in every replicate (Figure 1A). Type 2 isolate VR-2332 inhibited CpG-ODN induced IFN-α secretion by 34% and all other isolates, including highly virulent type 2 isolates SS144, MN184 and JA-1262, showed lower levels or no inhibitory activity (Figure 1A). Stated another way, CpG-ODN stimulated high levels of IFN-α secretion by pDC in the absence or presence of numerous PRRSV genotypes.

### Genotype 1 and 2 PRRSV induce IFN-α in pDC

**Figure 2:**
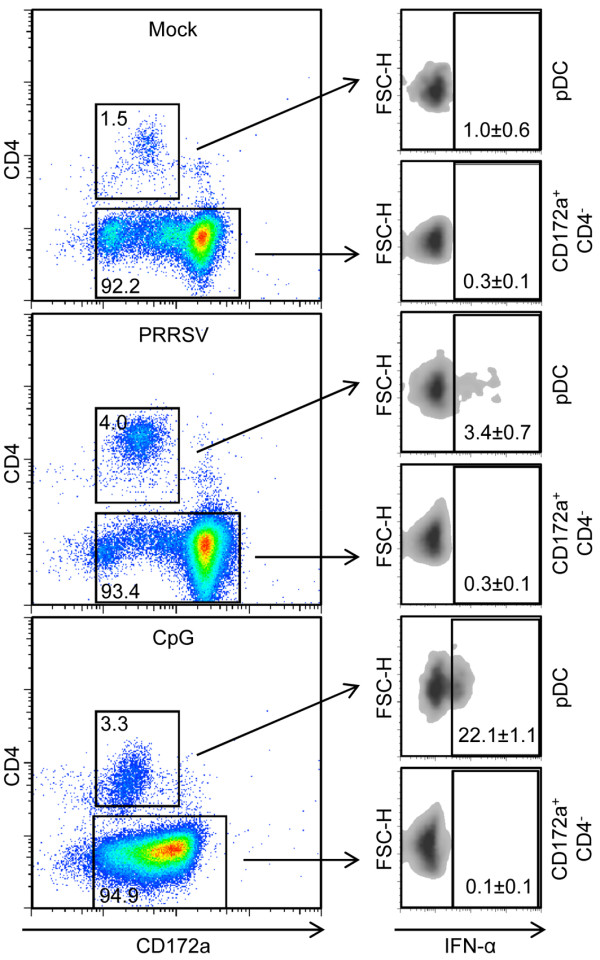
**IFN**-**α is induced by CD172**^**low**^**CD4**^**high **^**pDC.** Intracelluar staining of IFN-α was performed in CD172a enriched cells exposed to mock, PRRSV (MOI of 1 TCID_50_/cell) or CpG. Pseudo-color plot of pDC defined as CD172^low^CD4^high^ and CD172^+^CD4^-^ populations are gated (left panel) and density plot show that only gated pDC are positive for intracellular IFN-α (right panel) after PRRSV or CpG stimulation. Gate frequency is indicated as mean ± 1 standard deviation of one experiment performed in triplicate.

Since both type 1 and type 2 PRRSV isolates were not strongly suppressive, we analyzed their ability to directly activate pDC secretion of IFN-α. Incubation of CD172a^+^ cells with LV or its MARC-145 cell-adapted form, LVP23, at an MOI of 0.1 did not elicit reproducible IFN-α expression (data not shown), but showed robust IFN-α production at an MOI of 1 (Figure 1B). To further investigate if pDC production of IFN-α was a universal response to PRRSV, type 2 strains VR-2332, JA-1262, a virulent recent USA field isolate, SY0608, a highly pathogenic field isolate from China, and the avirulent type 1 Olot/91 strain were incubated with enriched pDC. All isolates directly elicited IFN-α secretion by pDC, with type 2 PRRSV prototype VR-2332 displaying the lowest activity, whereas type 1 strain Olot/91 showing the highest average effect (Figure 1C). Overall, all isolates induced IFN-α secretion and the range of IFN-α production was independent of genotype or isolate virulence. To assess whether monocytes could be involved in the PRRSV-induced IFN-α secretion, we incubated unsorted PBMCs, CD14^+^, CD14^+^CD4^-^ and CD14^-^CD4^+^ sorted cells with LV, LVP23, and CpG-ODN as a positive control. Whereas no or only low levels of IFN-α were found in PBMCs after PRRSV exposure (Figure 1D), high amounts were detected in the CD14^-^CD4^+^ cell fraction (Figure 1E). No IFN-α was detected in CD14^+^ cells (data not shown) excluding the possibility that monocytes induce IFN-α in response to PRRSV. To confirm that pDC were indeed the source of IFN-α in enriched CD172a cells, CD4, CD172a and intracellular IFN-α staining was performed. It revealed that only a proportion of CD4^+^ cells among the CD172a^+^ fraction were IFN-α positive after PRRSV or CpG-ODN stimulation whereas no IFN-α expressing cells were observed in the CD172^+^CD4^-^ cells (Figure 2). Together with the data shown in Figure 1E, these results demonstrate that CD172a^+^CD14^-^CD4^+^ pDC [23] are the source of PRRSV-derived IFN-α responses. We also observed a higher percentage of pDC when they were stimulated by PRRSV (4%) compared to mock (1.5%) after 16 h of culture suggesting that PRRSV promotes survival of the pDC. It confirms that PRRSV interacted with pDC and promoted IFN-α secretion since the frequency of mock-treated pDC decreased in absence of stimulus.

### PRRSV sensing by pDC does not require live virus and is mediated via TLR7

**Figure 3:**
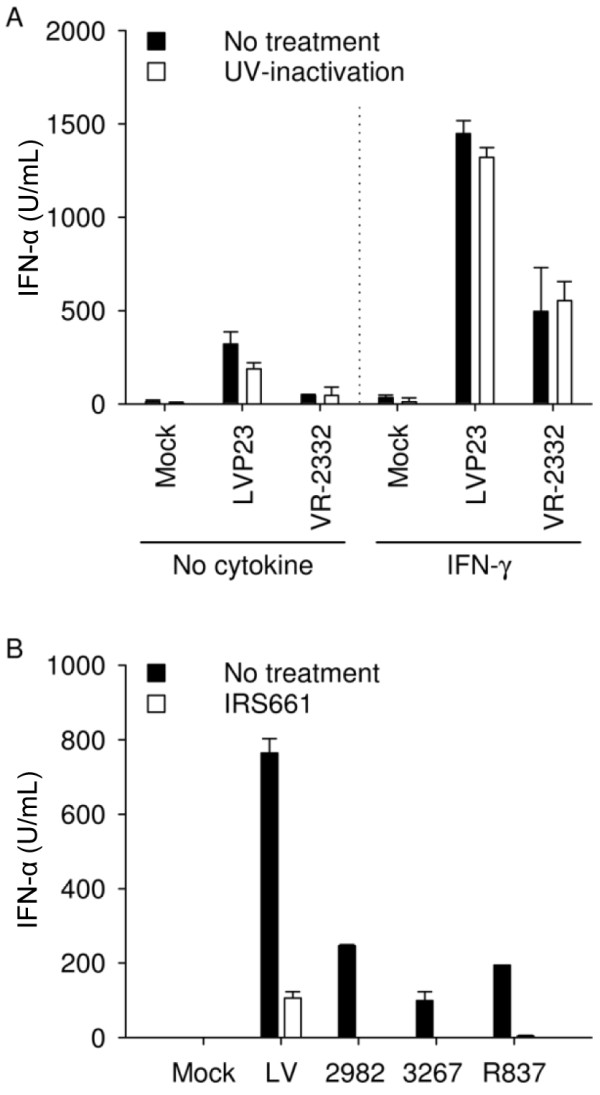
**PRRSV sensing by pDC does not require live virus and is mediated via TLR7.** (**A**) IFN-α induced by type 1 and 2 PRRSV (MOI of 1 TCID_50_/cell) in enriched pDC does not require live virus and is potentiated synergistically by IFN-γ. (**B**) Production of IFN-α by enriched pDC exposed to PRRSV (MOI of 2.5 TCID_50_/cell) is inhibited by IRS661, a TLR7 antagonist. IFN-α was determined by ELISA in supernatants harvested after 20 h. Bars indicate biological triplicates ± 1 standard deviation in one representative experiment out of two.

**Figure 4:**
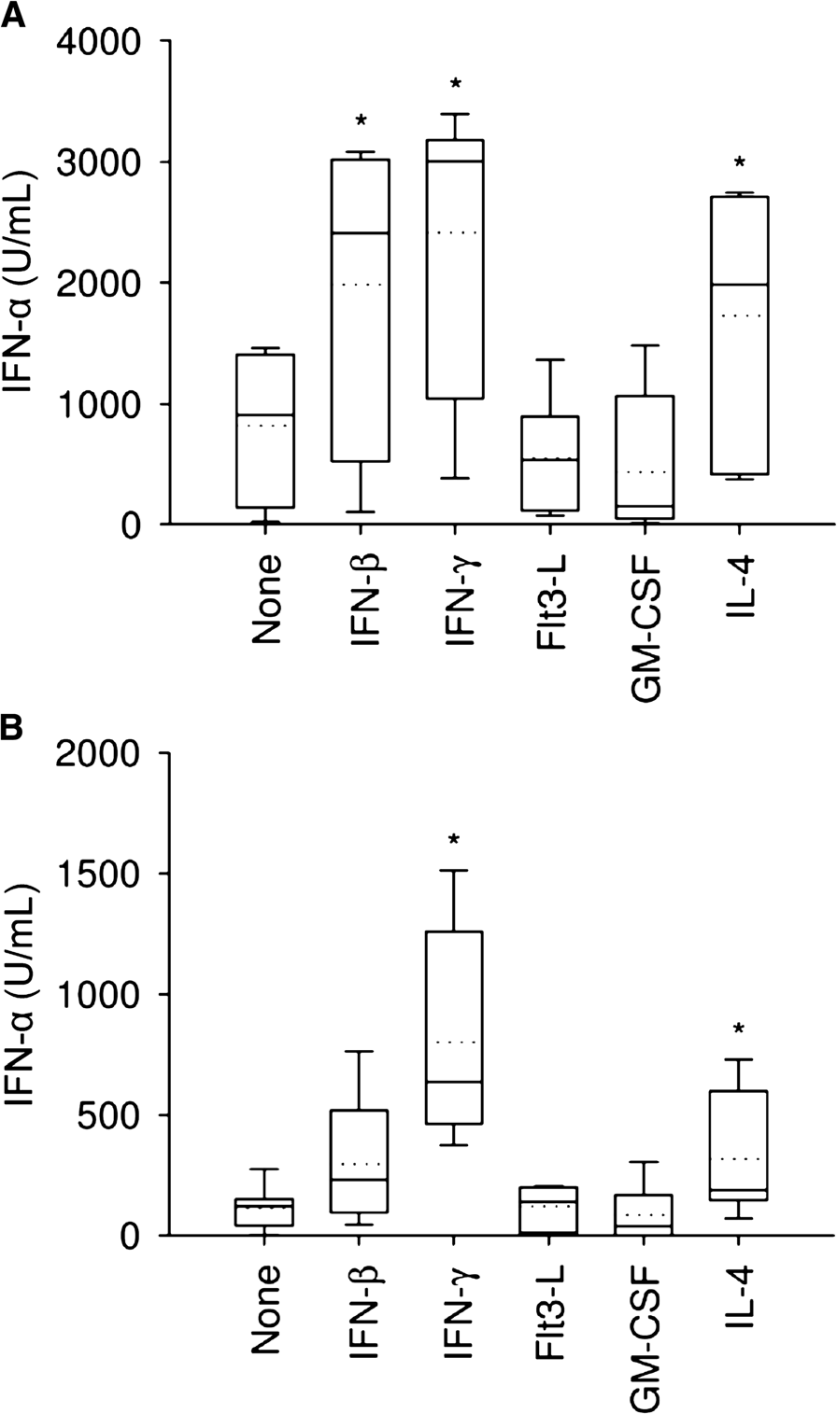
**Cytokine**-**enhancement of pDC IFN**-**α production induced by type 1 and 2 PRRSV.** LVP23 (**A**) or VR-2332 (**B**) at an MOI of 1 TCID_50_/cell were incubated with pDC alone or in the presence of IFN-β (100 U/mL), IFN-γ (10 ng/mL), Flt3-L (100 U/mL), GM-CSF (100 U/mL) and IL-4 (100 U/mL). IFN-α was determined by ELISA in supernatants harvested after 20 h. Boxplots are marked by the median (middle line), 25^th^ and 75^th^ percentiles (boxes), maximum and minimum values (whiskers) and the mean (dotted line) representing three independent experiments each performed in triplicate cultures. Significant difference in the median value is indicated by an asterisk mark compared to untreated cells (“none”) using the Mann–Whitney Rank Sum test (*P* < 0.02).

UV-inactivated type 1 LVP23 and type 2 VR-2332 PRRSV were used to evaluate if PRRSV-induced IFN-α responses in enriched pDC required live virus. As shown in Figure 3A, the intensity of IFN-α production was not altered by UV-inactivation indicating that the pDC response did not require replicating PRRSV. In the presence of IFN-γ, pDC-derived IFN-α secretion was increased with both UV-untreated and UV-treated PRRSV (see also Figure 4). Considering the central role of TLR7 in sensing RNA viruses by pDC [39], we investigated the effects of the specific TLR7 inhibitor IRS661 [40] to inhibit PRRSV-induced IFN-α production. IRS661 is active on porcine cells and inhibits influenza virus-mediated pDC activation compared to a scrambled oligonulceotide [37]. We observed that pDC-derived IFN-α responses were drastically reduced or abrogated by TLR7 inhibition (Figure 3B), indicating that the TLR7 pathway is intimately involved in pDC sensing of PRRSV.

### Host factors enhancing PRRSV-induced IFN-α responses by pDC

The function of pDC may be influenced by a cytokine microenvironment in vivo. Therefore, we were interested to determine the impact of cytokines on PRRSV-induced pDC activation. Type I and II IFN, Flt3-L, GM-CSF and IL-4, were evaluated for modulation of IFN-α secretion. As shown in Figure 4A, LVP23-induced secretion of IFN-α was enhanced by IFN-β, IFN-γ and IL-4, whereas strain VR-2332 induced production was enhanced by IFN-γ and IL-4 (Figure 4B). These results show that cytokines known to modulate macrophage function were able to promote pDC responses to PRRSV.

### PRRSV does not infect pDC

**Figure 5:**
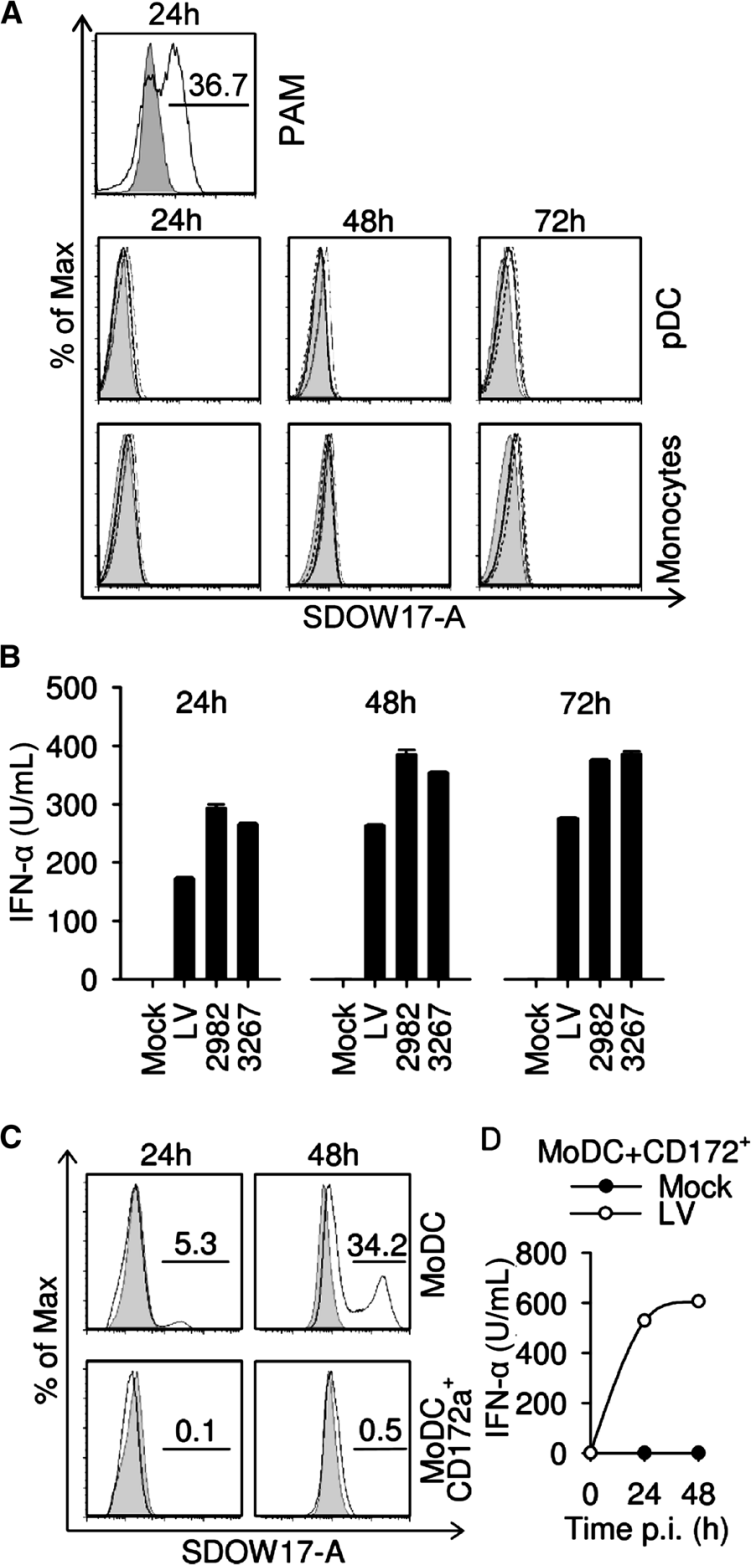
**pDC are resistant to PRRSV infection and can prevent infection of MoDC.** (**A**) Nucleocapsid expression in CD172a^low^CD4^+^ pDC and CD172a^high^CD4^-^ monocytes at 24, 48 and 72 h post infection with LV (black line histogram), 2982 (dotted line) or 3267 (dashed line) at an MOI of 2.5 TCID_50_/cell. Mock-treated cell fluorescence is shown as grey filled histograms. PAM infected with LV (MOI of 1 TCID_50_/cell) was used as positive control (upper panel). (**B**) IFN-α levels measured in the cultures shown in A. Bars represent means of triplicate cultures ± 1 standard deviation. A representative example from three independent experiments is shown. (**C**) Detection of PRRSV nucleocapsid in MoDC cultured alone or with enriched pDC at 24 and 48 h post infection with LV. One million MoDC co-cultured with one millions of enriched pDC or two millions MoDC alone were exposed to LV at an MOI of 1 TCID_50_/cell. Histograms were obtained from forward/side scatter gated MoDC treated with mock control (tinted line) or LV (black line). (**D**) Time course of IFN-α secretion in co-cultures of MoDC and pDC shown in panel **C**. One representative experiment out of two is shown.

PRRSV infects macrophages, MoDC, and monocyte-derived macrophages in vitro [6,8,9]. However, sorted CD172a^+^ cell populations of pDC were not permissive to infection. Flow cytometric three-colour analysis did not reveal the presence PRRSV nucleocapsid in gated pDC or monocytes even after prolonged incubation with PRRSV for three days (Figure 5A). These results are consistent with a previous report employing GFP tagged PRRSV [26], indicating that pDC are not permissive to PRRSV. High levels of IFN-α were detected in these cultures, confirming that productive infection is not required for pDC sensing of PRRSV (Figure 5B).

### pDC protect MoDCs from PRRSV infection

PRRSV appears to be highly sensitive to type I IFN [12-15]. Thus, we examined if IFN-α production by pDC was able to protect permissive cells from PRRSV infection. MoDC were cultured with PRRSV alone or in the presence of enriched pDC. While MoDC alone were infected by PRRSV, it was prevented in the presence of enriched pDC (Figure 5C). Figure 5D shows that IFN-α was present in co-culture supernatants, but not in the supernatant of MoDC alone (data not shown). It must be noted that IFN-α is efficient to limit PRRSV replication in MoDC [12] however other important proteins and antiviral factors such as IFN-β might contribute to protection of the permissive MoDC against PRRSV.

## Discussion

In contrast to previous studies which showed that genotype 2 PRRSV are potent antagonists of type I IFN responses in pDC [26], we demonstrate that neither type 1 nor type 2 PRRSV isolates strongly inhibit CpG-ODN-induced IFN-α by enriched pDC. Furthermore, the prototype type 2 strain VR-2332, although more inhibitory than the genotype 1 viruses tested, was not as inhibitory as previously reported [26]. Indeed, porcine pDC respond positively to the presence of viable or inactivated PRRSV by secretion of IFN-α in amounts sufficient to protect permissive MoDC from PRRSV infection. Differences in animal age were not important, as we obtained similar results with 6 week-old and 1 year-old blood donors. However, the genetic of the pigs could possibly play a role since differential expression genes, especially *IFNA*, have been highlighted in phenotypic pig groups in response to PRRSV [41]. Another difference compared to the data from Calzada-Nova et al. could be the methodology and cell isolation procedures. Interestingly, the highly pathogenic Chinese isolate was the most suppressive virus tested, pointing to possible strain-dependent differences in the interaction of PRRSV with pDC which should be further investigated with similarly pathogenic viruses. The strain differences in the suppression of IFN-α might relate to certain PRRSV proteins that counteract downstream elements of the pathway [42].

By this work, we also demonstrated that both type 1 and type 2 PRRSV isolates induce pDC-derived IFN-α production which is mainly triggered by the TLR7 signaling pathway. Compared to other viruses, including TGEV [43,44], influenza virus [45], classical swine fever virus (CSFV) [46] and foot-and-mouth disease virus (FMDV) [37,47], tested in our laboratory using the same methodology, the levels of IFN-α induced by PRRSV could be classified as moderate. PRRSV is clearly a stronger inducer than CSFV or FMDV, but less potent than TGEV or influenza virus, which reach the same levels as CpG-ODN. The responses observed in vitro are relevant to in vivo conditions, in which systemic IFN-α or IFN-α secreting cells in the lung were reported in several pigs infected with various PRRSV isolates [15,16,19,20,48]. Also, Barranco et al. reported increased numbers of non-identified IFN-α expressing cells by immunohistochemistry in lymph nodes of animals infected with the Spanish isolate 2982 [49]. Interestingly, the ability of PRRSV to induce IFN-α does not seem to be related to the suppression of TLR9 ligand-induced IFN-α. The highly pathogenic Chinese strain, SY0608, induced similar levels of IFN-α as did LVP23, but was more suppressive for CpG-induced IFN-α compared to all strains tested.

Host factors regulating pDC responses may be critical to anti-PRRSV responses, since the presence of different patterns of cytokines could promote an immunological micro-environment favoring or inhibiting pDC responses. For example, in mice Th1-matured pDC stimulated by TLR7 ligand respond more strongly with higher secretion of IL-6 and IFN-α than do Th2-pDC, but both Th1- and Th2-pDC were more strongly activated compared to unpolarized pDC [50]. As we show here in swine, both IFN-γ and IL-4 enhance pDC responses to PRRSV. IFN-β only enhanced pDC responsiveness to genotype 1 PRRSV; it is not yet known if this is a general feature of type 1 PRRSV or a strain-specific effect. The regulatory cytokine IL-10 was thought to strongly impact PRRSV pathogenesis and was one of the proposed mechanisms used by PRRSV to escape the innate and adaptive immune system. However, the induction of IL-10 by PRRSV is not clearly established [11,51], although its presence suppresses type I IFN induction in pDC [52,53]. It has been reported that PRRSV could induce IL-4 in the serum [20] and IFN-γ both in the serum [54] and in the lungs [48] of infected pigs. In particular IFN-γ could play multiple beneficial roles against PRRSV. It is known to have an antiviral activity against PRRSV [55], to generally promote natural killer and cytotoxic T-cell activity and to induce classical macrophages activation with increased anti-microbial functions, antigen-presenting activities and reduced ability to secrete regulatory cytokines such as IL-10 [56]. As IFN-α is known to promote IFN-γ, our data showing that vice versa IFN-γ also promotes IFN-α in pDC, it would appear that stimulating this axis will be beneficial for immunity against PRRSV. IL-4 on the other hand is known to promote the alternative pathway of macrophage activation resulting in “wound-healing” macrophages [56], which would be important to repair tissue damage in the lung during PRRSV infection. Through promoting pDC activation by PRRSV, this cytokine would also help establishing an antiviral state.

The presence of pDC prevented PRRSV infection and killing of MoDC, and thereby indirectly promoting cross-presentation of PRRSV antigens derived from apoptotic infected lung macrophages. This effect may be caused not only by IFN-α but also by other type I IFN’s such as IFN-β, known to be produced by pDC [57]. In addition to its antiviral activity, IFN-α is an efficient natural adjuvant promoting adaptive immune responses [15,58]. For these reasons pDC responses induced by PRRSV are relevant to understanding antiviral immune responses and the pathogenesis of PRRS.

## Competing interests

The authors declare that they have no competing interests.

## Authors’ contributions

AB and AS designed experiments. Experimental work performed by AB. EM and MM provided the viruses employed. AB, AS, EM and MM contributed to the manuscript preparation, revision and provided important intellectual input. All authors read and approved the final manuscript.

## References

[B1] Perry AK Chen G Zheng D Tang H Cheng G The host type I interferon response to viral and bacterial infections Cell Res 2005 15 407 422 10.1038/sj.cr.7290309 15987599

[B2] Sadler AJ Williams BR Interferon-inducible antiviral effectors Nat Rev Immunol 2008 8 559 568 10.1038/nri2314 18575461 PMC2522268

[B3] Haller O Kochs G Weber F The interferon response circuit: induction and suppression by pathogenic viruses Virology 2006 344 119 130 10.1016/j.virol.2005.09.024 16364743 PMC7125643

[B4] Murtaugh MP Xiao Z Zuckermann F Immunological responses of swine to porcine reproductive and respiratory syndrome virus infection Viral Immunol 2002 15 533 547 10.1089/088282402320914485 12513925

[B5] Murtaugh MP Stadejek T Abrahante JE Lam TT Leung FC The ever-expanding diversity of porcine reproductive and respiratory syndrome virus Virus Res 2010 154 18 30 10.1016/j.virusres.2010.08.015 20801173

[B6] Duan X Nauwynck HJ Pensaert MB Effects of origin and state of differentiation and activation of monocytes/macrophages on their susceptibility to porcine reproductive and respiratory syndrome virus (PRRSV) Arch Virol 1997 142 2483 2497 10.1007/s007050050256 9672608 PMC7086874

[B7] Xiao Z Batista L Dee S Halbur P Murtaugh MP The level of virus-specific T-cell and macrophage recruitment in porcine reproductive and respiratory syndrome virus infection in pigs is independent of virus load J Virol 2004 78 5923 5933 10.1128/JVI.78.11.5923-5933.2004 15140990 PMC415812

[B8] Silva-Campa E Cordoba L Fraile L Flores-Mendoza L Montoya M Hernandez J European genotype of porcine reproductive and respiratory syndrome (PRRSV) infects monocyte-derived dendritic cells but does not induce Treg cells Virology 2010 396 264 271 10.1016/j.virol.2009.10.024 19913865

[B9] Vincent AL Thacker BJ Halbur PG Rothschild MF Thacker EL In vitro susceptibility of macrophages to porcine reproductive and respiratory syndrome virus varies between genetically diverse lines of pigs Viral Immunol 2005 18 506 512 10.1089/vim.2005.18.506 16212529

[B10] Yoo D Song C Sun Y Du Y Kim O Liu HC Modulation of host cell responses and evasion strategies for porcine reproductive and respiratory syndrome virus Virus Res 2010 154 48 60 10.1016/j.virusres.2010.07.019 20655963 PMC7114477

[B11] Darwich L Diaz I Mateu E Certainties, doubts and hypotheses in porcine reproductive and respiratory syndrome virus immunobiology Virus Res 2010 154 123 132 10.1016/j.virusres.2010.07.017 20659507

[B12] Loving CL Brockmeier SL Sacco RE Differential type I interferon activation and susceptibility of dendritic cell populations to porcine arterivirus Immunology 2007 120 217 229 10.1111/j.1365-2567.2006.02493.x 17116172 PMC2265861

[B13] Sang Y Rowland RR Blecha F Porcine type I interferons: polymorphic sequences and activity against PRRSV BMC Proc 2011 5 Suppl 4 S8 10.1186/1753-6561-5-S4-S8 21645323 PMC3108238

[B14] Luo R Fang L Jin H Jiang Y Wang D Chen H Xiao S Antiviral activity of type I and type III interferons against porcine reproductive and respiratory syndrome virus (PRRSV) Antiviral Res 2011 91 99 101 10.1016/j.antiviral.2011.04.017 21569798

[B15] Brockmeier SL Loving CL Nelson EA Miller LC Nicholson TL Register KB Grubman MJ Brough DE Kehrli ME Jr The presence of alpha interferon at the time of infection alters the innate and adaptive immune responses to porcine reproductive and respiratory syndrome virus Clin Vaccine Immunol 2012 19 508 514 10.1128/CVI.05490-11 22301694 PMC3318278

[B16] Albina E Carrat C Charley B Interferon-alpha response to swine arterivirus (PoAV), the porcine reproductive and respiratory syndrome virus J Interferon Cytokine Res 1998 18 485 490 10.1089/jir.1998.18.485 9712364

[B17] Lee SM Schommer SK Kleiboeker SB Porcine reproductive and respiratory syndrome virus field isolates differ in in vitro interferon phenotypes Vet Immunol Immunopathol 2004 102 217 231 10.1016/j.vetimm.2004.09.009 15507307 PMC7112598

[B18] Zhang H Guo X Nelson E Christopher-Hennings J Wang X Porcine reproductive and respiratory syndrome virus activates the transcription of interferon alpha/beta (IFN-alpha/beta) in monocyte-derived dendritic cells (Mo-DC) Vet Microbiol 2012 159 494 498 10.1016/j.vetmic.2012.04.025 22592217 PMC7127654

[B19] Liu Y Shi W Zhou E Wang S Hu S Cai X Rong F Wu J Xu M Li L Dynamic changes in inflammatory cytokines in pigs infected with highly pathogenic porcine reproductive and respiratory syndrome virus Clin Vaccine Immunol 2010 17 1439 1445 10.1128/CVI.00517-09 20631336 PMC2944458

[B20] Dwivedi V Manickam C Binjawadagi B Linhares D Murtaugh MP Renukaradhya GJ Evaluation of immune responses to porcine reproductive and respiratory syndrome virus in pigs during early stage of infection under farm conditions Virol J 2012 9 45 10.1186/1743-422X-9-45 22340040 PMC3298799

[B21] Liu YJ IPC: professional type 1 interferon-producing cells and plasmacytoid dendritic cell precursors Annu Rev Immunol 2005 23 275 306 10.1146/annurev.immunol.23.021704.115633 15771572

[B22] Colonna M Trinchieri G Liu YJ Plasmacytoid dendritic cells in immunity Nat Immunol 2004 5 1219 1226 10.1038/ni1141 15549123

[B23] Summerfield A McCullough KC The porcine dendritic cell family Dev Comp Immunol 2009 33 299 309 10.1016/j.dci.2008.05.005 18582937 PMC7103208

[B24] Guzylack-Piriou L Alves MP McCullough KC Summerfield A Porcine Flt3 ligand and its receptor: generation of dendritic cells and identification of a new marker for porcine dendritic cells Dev Comp Immunol 2010 34 455 464 10.1016/j.dci.2009.12.006 20015454

[B25] Calzada-Nova G Schnitzlein W Husmann R Zuckermann FA Characterization of the cytokine and maturation responses of pure populations of porcine plasmacytoid dendritic cells to porcine viruses and toll-like receptor agonists Vet Immunol Immunopathol 2010 135 20 33 10.1016/j.vetimm.2009.10.026 19939462 PMC7126865

[B26] Calzada-Nova G Schnitzlein WM Husmann RJ Zuckermann FA North American porcine reproductive and respiratory syndrome viruses inhibit type I interferon production by plasmacytoid dendritic cells J Virol 2011 85 2703 2713 10.1128/JVI.01616-10 21191013 PMC3067927

[B27] Wensvoort G Terpstra C Pol JM ter Laak EA Bloemraad M de Kluyver EP Kragten C van Buiten L den Besten A Wagenaar F Broekhuijsen JM Moonen PLJM Zetstra T de Boer EA Tibben HJ de Jong MF van ‘t Veld P Greenland GJR van Gennep JA Voets MT Verheijden JHM Braamskamp J Mystery swine disease in The Netherlands: the isolation of Lelystad virus Vet Q 1991 13 121 130 10.1080/01652176.1991.9694296 1835211

[B28] Gimeno M Darwich L Diaz I de la Torre E Pujols J Martin M Inumaru S Cano E Domingo M Montoya M Mateu E Cytokine profiles and phenotype regulation of antigen presenting cells by genotype-I porcine reproductive and respiratory syndrome virus isolates Vet Res 2011 42 9 10.1186/1297-9716-42-9 21314968 PMC3037899

[B29] Collins JE Benfield DA Christianson WT Harris L Hennings JC Shaw DP Goyal SM McCullough S Morrison RB Joo HS Gorcyca D Chladek D Isolation of swine infertility and respiratory syndrome virus (isolate ATCC VR-2332) in North America and experimental reproduction of the disease in gnotobiotic pigs J Vet Diagn Invest 1992 4 117 126 10.1177/104063879200400201 1616975

[B30] Li Y Wang X Bo K Tang B Yang B Jiang W Jiang P Emergence of a highly pathogenic porcine reproductive and respiratory syndrome virus in the Mid-Eastern region of China Vet J 2007 174 577 584 10.1016/j.tvjl.2007.07.032 17869553

[B31] Basta S Carrasco CP Knoetig SM Rigden RC Gerber H Summerfield A McCullough KC Porcine alveolar macrophages: poor accessory or effective suppressor cells for T-lymphocytes Vet Immunol Immunopathol 2000 77 177 190 10.1016/S0165-2427(00)00237-3 11137117

[B32] Carrasco CP Rigden RC Schaffner R Gerber H Neuhaus V Inumaru S Takamatsu H Bertoni G McCullough KC Summerfield A Porcine dendritic cells generated in vitro: morphological, phenotypic and functional properties Immunology 2001 104 175 184 10.1046/j.1365-2567.2001.01299.x 11683958 PMC1783296

[B33] Guzylack-Piriou L Balmelli C McCullough KC Summerfield A Type-A CpG oligonucleotides activate exclusively porcine natural interferon-producing cells to secrete interferon-alpha, tumour necrosis factor-alpha and interleukin-12 Immunology 2004 112 28 37 10.1111/j.1365-2567.2004.01856.x 15096181 PMC1782461

[B34] McCullough KC Schaffner R Fraefel W Kihm U The relative density of CD44-positive porcine monocytic cell populations varies between isolations and upon culture and influences susceptibility to infection by African swine fever virus Immunol Lett 1993 37 83 90 10.1016/0165-2478(93)90136-P 8225410

[B35] Husser L Alves MP Ruggli N Summerfield A Identification of the role of RIG-I, MDA-5 and TLR3 in sensing RNA viruses in porcine epithelial cells using lentivirus-driven RNA interference Virus Res 2011 159 9 16 10.1016/j.virusres.2011.04.005 21539869

[B36] Summerfield A Horn MP Lozano G Carrasco CP Atze K McCullough K C-kit positive porcine bone marrow progenitor cells identified and enriched using recombinant stem cell factor J Immunol Methods 2003 280 113 123 10.1016/S0022-1759(03)00273-4 12972192

[B37] Lannes N Python S Summerfield A Interplay of foot-and-mouth disease virus, antibodies and plasmacytoid dendritic cells: virus opsonization under non-neutralizing conditions results in enhanced interferon-alpha responses Vet Res 2012 43 64 10.1186/1297-9716-43-64 22934974 PMC3479418

[B38] Diaz De Arce H Artursson K L'Haridon R Perers A La Bonnardiere C Alm GV A sensitive immunoassay for porcine interferon-alpha Vet Immunol Immunopathol 1992 30 319 327 10.1016/0165-2427(92)90102-V 1372135

[B39] Gilliet M Cao W Liu YJ Plasmacytoid dendritic cells: sensing nucleic acids in viral infection and autoimmune diseases Nat Rev Immunol 2008 8 594 606 10.1038/nri2358 18641647

[B40] Pawar RD Ramanjaneyulu A Kulkarni OP Lech M Segerer S Anders HJ Inhibition of Toll-like receptor-7 (TLR-7) or TLR-7 plus TLR-9 attenuates glomerulonephritis and lung injury in experimental lupus J Am Soc Nephrol 2007 18 1721 1731 10.1681/ASN.2006101162 17460144

[B41] Arceo ME Ernst CW Lunney JK Choi I Raney NE Huang T Tuggle CK Rowland RR Steibel JP Characterizing differential individual response to porcine reproductive and respiratory syndrome virus infection through statistical and functional analysis of gene expression Front Genet 2012 3 321 23335940 10.3389/fgene.2012.00321 PMC3546301

[B42] Sun Y Han M Kim C Calvert JG Yoo D Interplay between interferon-mediated innate immunity and porcine reproductive and respiratory syndrome virus Viruses 2012 4 424 446 10.3390/v4040424 22590680 PMC3347317

[B43] Charley B Lavenant L Characterization of blood mononuclear cells producing IFN alpha following induction by coronavirus-infected cells (porcine transmissible gastroenteritis virus) Res Immunol 1990 141 141 151 10.1016/0923-2494(90)90133-J 2167506 PMC7135751

[B44] Summerfield A Guzylack-Piriou L Schaub A Carrasco CP Tache V Charley B McCullough KC Porcine peripheral blood dendritic cells and natural interferon-producing cells Immunology 2003 110 440 449 10.1111/j.1365-2567.2003.01755.x 14632641 PMC1783075

[B45] Bel M Ocana-Macchi M Liniger M McCullough KC Matrosovich M Summerfield A Efficient sensing of avian influenza viruses by porcine plasmacytoid dendritic cells Viruses 2011 3 312 330 10.3390/v3040312 21994734 PMC3185703

[B46] Fiebach AR Guzylack-Piriou L Python S Summerfield A Ruggli N Classical swine fever virus N(pro) limits type I interferon induction in plasmacytoid dendritic cells by interacting with interferon regulatory factor 7 J Virol 2011 85 8002 8011 10.1128/JVI.00330-11 21680532 PMC3147952

[B47] Guzylack-Piriou L Bergamin F Gerber M McCullough KC Summerfield A Plasmacytoid dendritic cell activation by foot-and-mouth disease virus requires immune complexes Eur J Immunol 2006 36 1674 1683 10.1002/eji.200635866 16783856

[B48] Gomez-Laguna J Salguero FJ Barranco I Pallares FJ Rodriguez-Gomez IM Bernabe A Carrasco L Cytokine expression by macrophages in the lung of pigs infected with the porcine reproductive and respiratory syndrome virus J Comp Pathol 2010 142 51 60 10.1016/j.jcpa.2009.07.004 19691969 PMC7126906

[B49] Barranco I Gomez-Laguna J Rodriguez-Gomez IM Quereda JJ Salguero FJ Pallares FJ Carrasco L Immunohistochemical expression of IL-12, IL-10, IFN-alpha and IFN-gamma in lymphoid organs of porcine reproductive and respiratory syndrome virus-infected pigs Vet Immunol Immunopathol 2012 149 262 271 10.1016/j.vetimm.2012.07.011 22889555

[B50] Bratke K Klein C Kuepper M Lommatzsch M Virchow JC Differential development of plasmacytoid dendritic cells in Th1- and Th2-like cytokine milieus Allergy 2011 66 386 395 10.1111/j.1398-9995.2010.02497.x 21039603

[B51] Klinge KL Vaughn EM Roof MB Bautista EM Murtaugh MP Age-dependent resistance to Porcine reproductive and respiratory syndrome virus replication in swine Virol J 2009 6 177 10.1186/1743-422X-6-177 19860914 PMC2773768

[B52] Lannes N Summerfield A Regulation of porcine plasmacytoid dendritic cells by cytokines PLoS Onein press 10.1371/journal.pone.0060893 PMC3620061 23577175

[B53] Duramad O Fearon KL Chan JH Kanzler H Marshall JD Coffman RL Barrat FJ IL-10 regulates plasmacytoid dendritic cell response to CpG-containing immunostimulatory sequences Blood 2003 102 4487 4492 10.1182/blood-2003-07-2465 12946990

[B54] Wesley RD Lager KM Kehrli ME Jr Infection with Porcine reproductive and respiratory syndrome virus stimulates an early gamma interferon response in the serum of pigs Can J Vet Res 2006 70 176 182 16850939 PMC1477926

[B55] Rowland RR Robinson B Stefanick J Kim TS Guanghua L Lawson SR Benfield DA Inhibition of porcine reproductive and respiratory syndrome virus by interferon-gamma and recovery of virus replication with 2-aminopurine Arch Virol 2001 146 539 555 10.1007/s007050170161 11338389 PMC7087212

[B56] Mosser DM Edwards JP Exploring the full spectrum of macrophage activation Nat Rev Immunol 2008 8 958 969 10.1038/nri2448 19029990 PMC2724991

[B57] Szubin R Chang WL Greasby T Beckett L Baumgarth N Rigid interferon-alpha subtype responses of human plasmacytoid dendritic cells J Interferon Cytokine Res 2008 28 749 763 10.1089/jir.2008.0037 18937549 PMC2956691

[B58] Cull VS Broomfield S Bartlett EJ Brekalo NL James CM Coimmunisation with type I IFN genes enhances protective immunity against cytomegalovirus and myocarditis in gB DNA-vaccinated mice Gene Ther 2002 9 1369 1378 10.1038/sj.gt.3301809 12365002

