# Correction: Impact of genotype 1 and 2 of porcine reproductive and respiratory syndrome viruses on interferon-α responses by plasmacytoid dendritic cells

**DOI:** 10.1186/1297-9716-44-74

**Published:** 2013-09-11

**Authors:** Arnaud Baumann, Enric Mateu, Michael P Murtaugh, Artur Summerfield

**Affiliations:** 1 Institute of Virology and Immunoprophylaxis (IVI), Sensemattstrasse 293, 3147 Mittelhäusern, Switzerland; 2 Graduate School for Cellular and Biomedical Sciences, University of Bern, Bern, Switzerland; 3 Centre de Recerca en Sanitat Animal (CReSA), UAB-IRTA, Campus de la Universitat Autònoma de Barcelona, 08193, Bellaterra, Barcelona, Spain; 4 Department of Veterinary and Biomedical Sciences, University of Minnesota, St. Paul, MN 55108, USA

## Correction

After the publication of our article [1], we noted that the porcine reproductive and respiratory syndrome virus (PRRSV) strain referred to as SY0608 was not referred to correctly.

The correct reference for this PRRSV strain is RVB-581 [2,3], not SY0608.

We apologise for this oversight and any inconvenience this may have caused.
